# Combinatorial Analysis of Secretory Immunoglobulin A (sIgA) Expression in Plants

**DOI:** 10.3390/ijms14036205

**Published:** 2013-03-18

**Authors:** Paloma Juarez, Estefania Huet-Trujillo, Alejandro Sarrion-Perdigones, Erica Elvira Falconi, Antonio Granell, Diego Orzaez

**Affiliations:** Institute of Molecular and Cellular Plant Biology (IBMCP), Spanish Research Council Agency (CSIC), Polytechnic University of Valencia (UPV), Avda Tarongers SN, Valencia 46022, Spain; E-Mails: pjuarez@ibmcp.upv.es (P.J.); eshuetru@upvnet.upv.es (E.H.-T.); asarrion@ibmcp.upv.es (A.S.-P.); erica_falconi@hotmail.com (E.E.F.); agranell@ibmcp.upv.es (A.G.)

**Keywords:** secretory IgA, antibody, rotavirus, GoldenBraid, plant synthetic biology

## Abstract

Delivery of secretory immunoglobulin A (sIgA) to mucosal surfaces as a passive immunotherapy agent is a promising strategy to prevent infectious diseases. Recombinant sIgA production in plants requires the co-expression of four transcriptional units encoding the light chain (LC), heavy chain (HC), joining chain (JC) and secretory component (SC). As a way to optimize sIgA production in plants, we tested the combinatorial expression of 16 versions of a human sIgA against the VP8* rotavirus antigen in *Nicotiana benthamiana*, using the recently developed GoldenBraid multigene assembly system. Each sIgA version was obtained by combining one of the two types of HC (α1 and α2) with one of the two LC types (k and λ) and linking or not a KDEL peptide to the HC and/or SC. From the analysis of the anti-VP8* activity, it was concluded that those sIgA versions carrying HCα1 and LCλ provided the highest yields. Moreover, ER retention significantly increased antibody production, particularly when the KDEL signal was linked to the SC. Maximum expression levels of 32.5 μg IgA/g fresh weight (FW) were obtained in the best performing combination, with an estimated 33% of it in the form of a secretory complex.

## 1. Introduction

Monoclonal antibodies (mAbs) have been used in research and diagnosis for many years, and their application in health is increasing rapidly. They play an essential role in cancer therapy [1], however topical and oral immunotherapy of mucosal surfaces with mAbs is also of great interest, as it can block the entry and transmission of human pathogens [2–4].

There are different expression systems available for the production of recombinant antibodies, each with its advantages and shortcomings. Plants are one of the most interesting platforms for recombinant antibody production, because they are cost-effective, highly scalable, have a low risk of contamination with mammalian pathogens [5–8] and also can perform post-translational modifications similar to mammals, as, for example, *N*- and *O*-glycosylation [9]. IgA is the most abundant antibody in mucus, and it forms part of the first line of defense against infectious agents. IgA can be present in the body fluids in its monomeric form (mIgA), containing only heavy chain (HC) and light chain (LC) or forming a secretory IgA (sIgA), a multiprotein structure comprising two full IgA molecules dimerized by a short joining chain (JC) and surrounded by the secretory component (SC), a polypeptide resulting from the proteolytic cleavage of the poly-immunoglobulin receptor (pIgR). IgA and, particularly, sIgA are good candidates for mucosal passive immunotherapy, having a number of advantages over IgG (e.g., the presence of four antigen binding sites, increased resistance against proteolysis in the gastrointestinal tract and the blocking of some bacterial pathogens mediated by carbohydrates, both in the HC and the SC) [2–4]. Despite this, most research effort on plant-made recombinant antibodies has been made on monoclonal IgG antibodies.

The first attempt to produce a plant-made sIgA for passive immunization was the murine hybrid Guy’s 13 sIgG/A [10,11], which was evaluated in phase I and II clinical trials [12] as CaroxR™. Since then, various groups have expressed sIgA in plants [13–16]. However, there is a lack of information about what structural requirements are the best to produce functional, fully human sIgA in plants with maximum activity.

There are a number of options available when designing a new antibody in sIgA format, which could lead to completely different products for the same purpose. Therefore, it is important to study how modifications in the engineered parts will affect antibody function. In this work, we focus on the study of the *in vitro* binding activity of the final product, although other functional considerations, such as *in vivo* activity or stability, could also be tested with an adequate setup. In the first place, there are several options concerning structural design: LC may occur in two isotype forms, designated kappa (κ) and lambda (λ), with no functional differences described between them [17], and two types of HC, namely the α1 and the α2. In particular, the hinge region differs significantly between the two HC isoforms. The hinge region of HCα1 is comprised of 23 residues, while HCα2 is made of only 10 residues. The greater number of amino-acids in the IgA α1 provides an extended structure and a greater antigenic reach, while IgA α2 is more compact and, therefore, less susceptible to proteolitic cleavage [18].

Secondly, subcellular localization may also affect the overall efficacy of the antibody. Targeting antibody chains to specific compartments in the plant cell can improve the stability, yield and/or downstream processing [19]. The secretory pathway appears to be the most convenient route for a correct antibody folding and assembly, due to the oxidizing environment of the endoplasmic reticulum (ER), the low abundance of proteases and the presence of molecular chaperones. Moreover, protein glycosylation occurs only in the endomembrane system [20].

Once in the secretory pathway, there are several possible options; for example, the antibody can be efficiently retrieved from the *cis*-Golgi back to the ER using a *C*-terminal H/KDEL retention signal or deposited in the vacuole [21]. Several antibodies have also been reported as apoplastic [21–24]; however, in some cases, retention in the ER leads to a yield improvement [25] and avoids plant complex glycosylation patterns that could cause an unwanted immune response [26,27].

An appropriate way to produce the most efficient antibody design is to perform a combinatorial analysis of several versions of sIgA and select those which accumulate at higher levels and/or present an improvement on stability and activity. With traditional DNA assembly systems, this can become tedious work. However, with new standard modular cloning tools, like GoldenBraid (GB) [28] or MoClo [29], to achieve all the combinations should be facilitated. These technologies open a new way for optimization of antibody production in plants by experimentally testing the best subcellular targeting and isotype combinations for the expression of a target antibody.

As a proof of this concept, we have followed a combinatorial approach to optimize the plant production of a sIgA version of an anti-rotavirus monoclonal antibody (2A1 sIgA). The variable regions of 2A1 antibody were initially selected by phage display against the VP8* peptide of the VP4 protein of the rotavirus SA11 strain capsid [30]. This antibody was previously described in its monomeric format, showing a strong rotavirus neutralization activity [31].

## 2. Results

### 2.1. GoldenBraid-Assisted Multigene Assembly of 16 Versions of Secretory IgA

**Figure 1:**
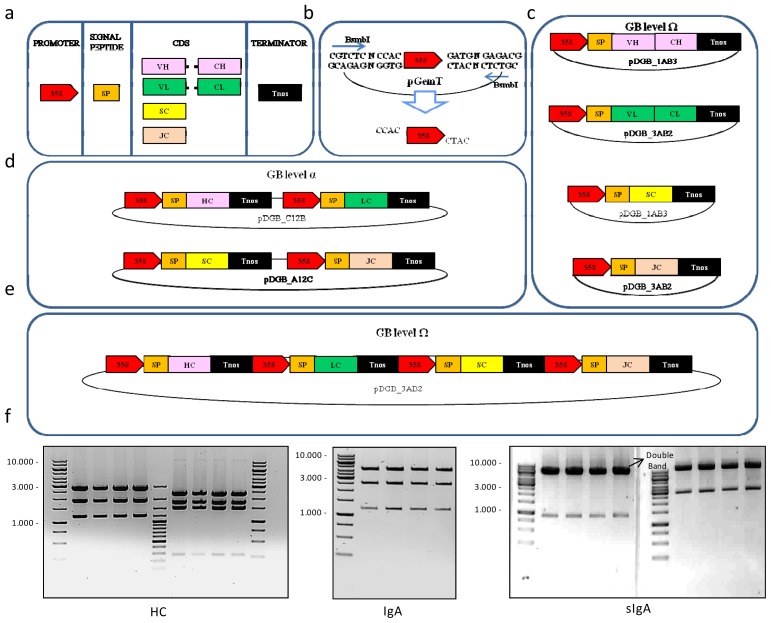
Assembly process of the secretory immunoglobulin A (sIgA). (**a**) Collection of basic parts necessary to construct a secretory IgA. Each basic part is cloned in a pGem-T vector. 35S, SP, VH-CH, VL-CL, SC, JC, Tnos, correspond, respectively, to the 35s CMV promoter, pectate lyase signal peptide, variable and constant regions of the heavy chain, variable and constant regions of the light chain, secretory component, J-chain and nopaline synthase terminator; (**b**) Example of domestication of a basic part. The 35s promoter is flanked by fixed BsmbI recognition-cleavage sites. The overhangs left by the BsmbI restriction enzyme converge with GB pDGB vectors on 5′ and on 3′, with the next basic part to assemble; (**c**) Multipartite assembly of the basic parts to form the four different transcriptional units: heavy chain (HC), light chain (LC), secretory component (SC) and J-chain (JC), into level Ω-GB destiny vectors (pDGB_1AB3 and pDGB_3AB2); (**d**) Binary assembly of transcriptional units in level α-GB destination vectors (pDGB_C12B and pDGB_A12C), in order to construct two different composite parts—IgA and JC-SC; (**e**) Last construct of sIgA by binary assembly of two composite parts in a final pDGB; (**f**) Example of restriction analysis of four colonies of each construct: left, BglII (expected bands of 2825, 1886 and 1197) and BglI (expected bands of 2345, 1790, 1498 and 275) restriction analysis of the HC transcriptional unit; middle, BglII (expected bands of 4183, 2495 and 1228 kDa) restriction analysis of IgA; right, BamHI (expected bands of 6815, 5857 and 913 kDa) and BsaI (expected bands of 10,664 + 2921 kDa) restriction analysis of sIgA.

A number of standard basic DNA pieces, named GBparts, were used as a starting point in the building of sIgA multigene structures. Basic GBparts comprised non-coding DNA regions, as the CaMV 35S constitutive promoter (35s) and the strong nopaline synthase transcription terminator (Tnos), together with a number of GB-adapted coding sequences required for the assembly of a functional sIgA, namely a signal peptide derived from the tomato pectate lyase gene (SP), the constant regions of the human HCα1 and HCα2, the constant regions of human LCλ and LCκ, the extracellular region of the human SC and the complete coding sequence of the human JC. In addition, the heavy and light variable regions of the anti-rotavirus VP8* peptide scFv antibody fragment 2A1 were also GB-adapted and incorporated to the GBpart collection (Figure 1a). GB-adaptation, also called GB-domestication, consisted of: (i) removal of internal BsaI and BsmBI restriction sites by PCR-directed mutagenesis; (ii) PCR-mediated addition of flanking GB standard bar-codes, which consist of standard labels of 11 nucleotides that serve to facilitate cloning and, ultimately, to specify the relative position of each GBpart in the assembly (see Figure 1b and Table S1); and (iii) TA-cloning of the resulting domesticated PCR products into pGEM-T vectors.

Standard modular cloning allows the combinatorial seamless assembly of synonymous GBparts. Thus, 16 versions of sIgA were constructed by combining the two types of HC (HCα1 and HCα2) with the two versions of LC (LCκ and LCλ) and retaining or not the complex in the endoplasmic reticulum by adding (or not) a *C*-terminal KDEL signal to the HC and/or SC (see Table 1). For the assembly process of the four genes in a single T-DNA, the GoldenBraid cloning system was used.

A number of steps were followed to construct each sIgA version. First, the so-called multipartite assembly reactions were performed. In multipartite reactions, GBparts are assembled together into binary destination vectors (Ω-GB vectors) to produce individual transcriptional units (TUs). To this end, GBparts are simply mixed together in a single tube with Ω-GB destination vector and subjected to a highly efficient restriction/ligation reaction, which will orderly assemble all the elements of the transcriptional unit together (Figure 1c). In GB multipartite assembly, properly assembled structures are distinguished from the initial destination plasmids using positive blue/white β-galactosidase selection. Multipartite assemblies comprising four individual GBparts (e.g., SC TUs) resulted in 95% white colonies on average, whereas 5-part GBpart assemblies (e.g., HC TUs) resulted in 85% white colonies on average. In all cases, four individual white colonies were selected and tested by restriction analysis and a minimum of three out of four colonies were found correct (see HC panel in Figure 1f as an example).

Once individual transcriptional units were constructed, they were assembled together into 2-part TUs structures using level α-GB destination vectors in a so-called binary GB reaction. Binary reactions combining LC + HC produced genetic modules for the expression of full monomeric IgA (mIgA), whereas SC and JC TUs were also combined in a single genetic module named SC-JC (Figure 1d). Last, mIgA and SC-JC composite parts were assembled together into a final level Ω-GB destination vector in order to construct the complete sIgA (Figure 1e). The final size of the sIgA constructs was of 13.6 kb. Binary assemblies ranged between 5% and 20% white colonies. For each construct, four colonies were selected for digestion, resulting in 100% correct colonies (see examples in Figure 1f).

### 2.2. Transient Expression in Nicotiana benthamiana of 16 Versions of sIgA against Rotavirus

All 16 binary plasmids containing sIgA versions against VP8* (see Table 1) were transferred to *Agrobacterium tumefaciens* and transiently transformed in *Nicotiana benthamiana* by means of agroinfiltration. For the resulting 16 plant samples, an initial screening was performed by antigen ELISA to detect anti-VP8* IgA activity in the clarified crude extracts of agroinfiltrated leaves using an anti-HC antibody for detection. To avoid potential proteolysis, protease inhibitor, PMSF, was added to every extract. In order to ensure the accuracy of the comparison among all the combinations, all samples were equalized on the basis of the luciferase activity of a cotransformed plasmid in which the nopaline synthase promoter drives the luciferase gene. Antigen ELISA tests showed high anti-VP8* binding activity in half of the samples (Table 1). Surprisingly, a very low activity was observed in all sIgA versions containing the LCκ. The integrity of kappa-sIgA constructs was confirmed by retro-transformation of *Agrobacterium* plasmids into *E. coli* and subsequent restriction analysis and sequencing. In addition, the low anti-VP8* activity was confirmed in a second ELISA experiment that yielded similar results (not shown). Consequently, work with LCκ versions was discontinued and all further analyses were done with the LCλ-containing sIgA versions.

**Figure 2:**
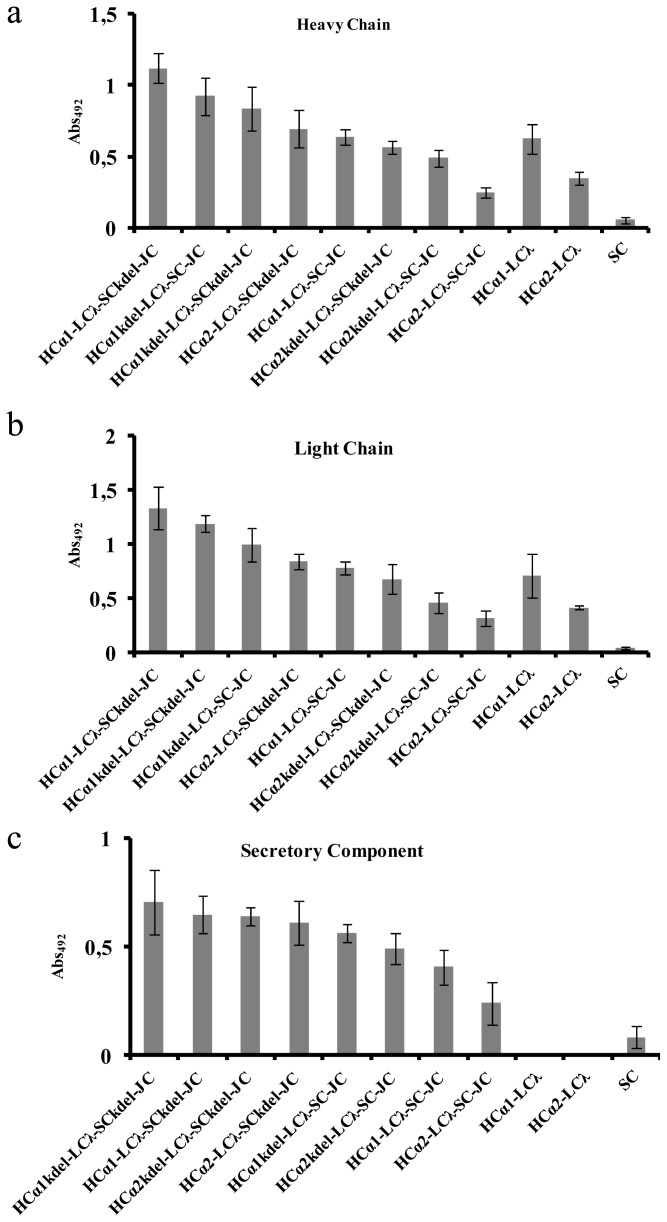
Combinatorial analysis of eight λ versions of sIgA by ELISA tests. (**a**) ELISA assay developed with anti-HC; (**b**) ELISA assay developed with anti-LCλ; (**c**) ELISA assay developed with anti-SC. All plates were coated with VP8* antigen. Three different leaves were infiltrated and tested for each sample. Means of the three biological replicates are represented, with error bars representing the standard deviation. All samples were equalized, with the luciferase reporter system as an internal standard.

A detailed examination of the remaining eight combinations was subsequently performed. The analysis was completed with TUs expressing monomeric IgA and free SC. Leaf age is a known factor influencing recombinant protein expression levels. When leaves of three different ages were transiently transformed and assayed for luciferase expression, a coefficient of variation of 22% was observed. Therefore, in order to increase the accuracy of the comparison, three independent leaves taken from different plants (leaves number 4, 5 and 6, counting from the base of the plant) were infiltrated per each construct. Each leaf was used as an individual biological replicate, and all results were subsequently normalized using a luciferase reporter system as an internal standard. The anti-VP8* activity of each combination was analyzed in detail by antigen-ELISA using three different detection tools, namely anti-HC, anti-LC and anti-SC antibodies. Antigen-ELISA tests against the HC were first carried out in order to give a first view of total IgA content (including mIgA and sIgA). Considerably high binding activity values were observed in all eight sIgA combinations, while the SC control remained negative. Figure 2a shows that ER retention had a positive effect, resulting in significantly higher anti-VP8* activity (*p*-value <0.01). This effect is more noticeable when the KDEL signal is added to the SC than to the HC, and the comparison between these two types of samples yielded also significant differences (*p*-value <0.01). The increase in anti-VP8* activity observed when the SC is targeted to the ER was the first evidence of the correct formation of a secretory complex. Interestingly, the ER-associated activity was shown to increase in a non-additive fashion, as the simultaneous targeting of both HC and SC to the ER did not yield significantly higher activity than individual targeting. A second interesting observation extracted from Figure 2a is that all the combinations containing HCα1 appear to be significantly more efficient than their HCα2 counterparts (*p*-value <0.01).

The above-described results were also confirmed using anti-LC as the detecting antibody, confirming the presence of LC in the functional complexes detected in the previous ELISA test. (Figure 2b).

Finally, antigen-ELISA tests using anti-SC as the detecting antibody were developed in order to specifically detect the anti-VP8* activity of sIgA complexes, as only SC-containing, VP8*-binding antibody complexes can be detected using this ELISA set up. Interestingly, the activity pattern observed for sIgA combinations was very similar to the one observed for total IgA using anti-HC (compare Figure 2a,c). In particular, the positive effect provided by the presence of a KDEL peptide in the SC was also observed using anti-SC detecting antibody, further confirming that the retention of the SC in the ER increases the overall anti-VP8* IgA activity by specifically stabilizing the sIgA subfraction.

Taking into account the whole analysis, the maximum anti-VP8* activity, detected both as total IgA and sIgA, was achieved with the HCα1-LCλ-JC-SCkdel combination. Among non-ER-retained forms, the maximum anti-VP8* activity was achieved with the HCα1-LCλ-JC-SC combination (Figure 2).

### 2.3. Detailed Characterization and Purification of the HCα1-LCλ-JC-SCkdel Combination

**Figure 3:**
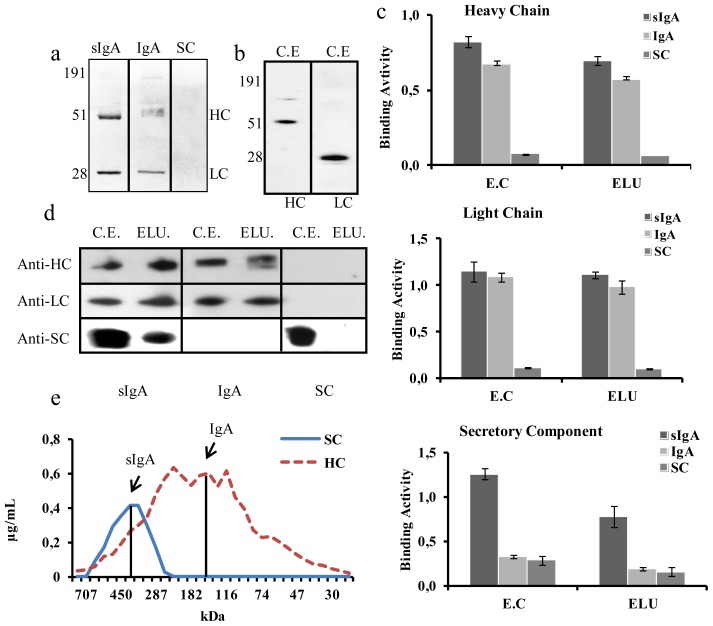
Characterization of SSL7-purified sIgA. (**a**) Coomassie stained SDS-PAGE under reducing conditions of elution fractions corresponding to HCα1-LCλ-JC-SCkdel (sIgA), HCα1kdel-LCλ (IgA) and free secretory component (SC) purification. Bands correspond to the heavy chain (HC) and light chain (LC); (**b**) Western blot analysis under reducing conditions of a clarified crude extract (CE) sample of HCα1kdel-LCλ (IgA) developed with anti-HC (left) and with anti-LC (right); (**c**) ELISA analysis, coated with VP8*, of starting clarified crude extract (CE) and elution fraction (ELU) of three samples: sIgA, IgA and free SC, performed as described in Figure 2. Upper panel developed with anti-HC, medium panel developed with anti-LC and lower panel developed with anti-SC. Means of three technical replicates are represented with error bars, indicating the standard deviation; (**d**) Western blot analysis under reducing conditions of the starting clarified crude extract (CE) and elution fractions (ELU) of three samples: sIgA, IgA and free SC. Upper lane developed with anti-HC, medium lane developed with anti-LC and lower lane developed with anti-SC; (**e**) ELISA analysis of fractions proceeding from gel filtration chromatography of a sample of SSL7-purified HCα1-LCλ-JC-SCkdel construct. Plates were coated with anti-HC and developed with HRP conjugated anti-HC (red) and anti-SC (blue). A standard curve of commercial IgA from human colostrum was obtained to calculate the concentration of IgA and sIgA (expressed as HC equivalents for proper comparison) for every fraction.

Once the best performing combination was identified, a detailed characterization of the resulting product was undertaken. *N. benthamiana* leaves were agroinfiltrated with the best sIgA-encoding multigenic construct (HCα1-LCλ-JC-SCkdel). A mIgA construct (HCα1kdel-LCλ) and a free secretory component construct (SC) were also agroinfiltrated to be used as controls. At 5 dpi, leaves were harvested, and crude extracts were clarified and used for analysis. The antibody content was quantified by sandwich ELISA using plates coated with anti-HC antibody. mIgA control was also analyzed by Western blot to assess the integrity of the HC and LC when transiently produced in plants, as shown in Figure 3b. It was anticipated that the agroinfiltration of HCα1-LCλ-JC-SCkdel would result in a mix of mIgA and sIgA. The total IgA content (calculated as HC equivalents) in clarified crude extracts was estimated by sandwich ELISA with an anti-HC detecting antibody, whereas an anti-SC detecting antibody was employed to estimate the sIgA content, using a standard curve made with sIgA from human colostrum. As shown in Table 2, both IgA and sIgA constructs yielded similar amounts of total IgA, estimated as HC equivalents, namely 31.6 ± 3.7 and 32.5 ± 1.1 μg/g fresh weight (FW) and representing 1.1% and 1.5% of the total soluble protein (TSP) in leaves, respectively. Expectedly, only background sIgA values were detected for the mIgA and SC-alone constructs, whereas the HCα1-LCλ-JC-SCkdel construct yielded 11.0 ± 0.2 μg/g FW of sIgA (expressed as equivalents of HC), corresponding to a 0.5 ± 0.1% TSP. According to these calculations, it was estimated that at least 33% of the total HC in HCα1-LCλ-JC-SCkdel-infiltrated leaves is present in the form of a sIgA complex (Table 2).

In principle, both mIgA and sIgA can be purified using SSL7-affinity chromatography, which binds the Cα2/Cα3 domain junction of the Fc (Fragment crystallizable) portion of the HC [32]. For affinity purification, clarified crude extracts were passed through SSL7-agarose columns. The purification steps were monitored by antigen-ELISA, coomassie stained SDS-PAGE and Western blot. Upon PAGE separation of the elution fractions, a 25 kDa band corresponding to the full size LC and a 55 kDa band corresponding to the full size HC were observable in coomassie stained gels from IgA and sIgA samples, after a single step of affinity chromatography (Figure 3a). The 64 kDa coomassie band that would correspond to the free SC was not detected by coomassie staining, but was readily detectable in Western Blot analysis from sIgA samples (Figure 3d). In contrast, SSL7 control purifications from SC-alone and mIgA agroinfiltrations did not yield any detectable SC band (Figure 3d).

The anti-VP8* binding activity of the three samples was also followed by antigen ELISA. ELISA plates were coated with VP8*, incubated with crude and SSL7 purified extracts and developed with anti-HC, anti-LC and anti-SC antibodies. As expected, anti-VP8* antibody formats containing HC and LC peptides were detected in crude and purified samples from IgA and sIgA agroinfiltrated constructs (Figure 3c), whereas anti-VP8* antibody formats containing SC were only observed in crude and purified samples derived from the sIgA construct. No SC was detected from SC-alone constructs, indicating that free-SC does not bind to VP8* nor SSL7. Interestingly, the relative content of SC-containing antibody seems to decline after SSL7 purification (compare EC and ELU fractions of sIgA sample in Figure 3c, lower panel). This decline is not observed when the same samples are tested for their HC and LC content. Indeed, quantification of purified samples confirmed these results. Of a total of 0.7 μg/mL of HC, only 0.1 μg/mL were in the form of sIgA, which accounts for 21%. This could indicate that at least part of the secretory complex present in the clarified crude extract is disassembled during the purification process, losing its SC peptide and yielding monomeric IgA structures.

With these results and in order to learn more about the proportion of sIgA in the total IgA, a gel filtration assay was performed. For this, sIgA from the best performing combination was purified and 500 μL of the elution loaded into a prepacked high-resolution gel filtration column with a separation range between 10 and 600 kDa. Fractions of 250 μL were collected and monitored by sandwich ELISA assays developed both with anti-HC and anti-SC. Figure 3d shows two partially overlapped peaks, the larger one corresponding to the total content of HC equivalents (including sIgA (371 kDa), mIgA (146 kDa) and possibly unfolded chains) and the smaller one developed with anti-SC, corresponding to 371 kDa sIgA (an estimated 20% of the total IgA content) (Figure 3e).

## 3. Discussion

The selection of the most appropriate isotype for recombinant antibody production in plants is rarely addressed using experimental approaches, as practical hurdles often override technical or functional considerations. Moreover, sIgA has been only occasionally considered as a feasible option for mucosal passive immunotherapy, despite its demonstrated appropriateness [10]. Even more, certain specific decisions concerning antibody design, as the choice for LC and HC isotypes or for subcellular localization, are rarely made on the basis of an exhaustive experimental analysis. However, it has been repeatedly observed that antibody expression levels diverge dramatically from case to case and that, e.g., the experimental selection of the most stable idiotypes, can bring considerable advantages in terms of yield [15,31].

A technical hurdle that compromises the selection among different antibody formats is the difficulty to produce multigenic structures that can be assayed in a combinatorial way. In the case of recombinant production of protein complexes in plants, the assembly of multiple transcriptional units in a single T-DNA is an inefficient and tedious task, particularly in the case of sIgA, which requires the co-expression of four TUs. As an alternative approach, some labs, including our own, have relied on *trans*- co-transformation, even though it implies reduced reproducibility and unwanted heterogeneity in the expression levels of the different proteins, often impeding the reach of solid conclusions [28]. Here, we demonstrate that the recently developed GB assembly system facilitates the combinatorial assembly of the four transcriptional units necessary for sIgA expression, ensuring the coordinated expression of the four genes in transient expression experiments.

In a first screening, it was determined that the use of LCκ severely reduces the activity levels of IgA 2A1. The LCκ used in this assay was codon-optimized for *Nicotiana benthamiana* expression, and therefore, inefficient codon-usage can be discarded as a possible cause for the observed expression. This observation was particularly surprising and, although the integrity of the constructs containing LCκ were exhaustively examined, we could not find any element in the genetic design that could account for the low activity observed in this set of samples. Provided that LCκ have been successfully employed before in the production of other plant-made antibodies [33,34], it is quite possible that this is a specific feature of the 2A1 variable region. If so, this would highlight the importance of combinatorial screenings for successful plant-made antibody expression. Further experiments using variable regions other than 2A1 will serve to discern if the low activity of kappa light chain is an isotype or idiotype-specific feature.

Detailed examination of the remaining combinations led to a number of conclusions. First, these experiments served as a demonstration that a fully human sIgA complex is formed in the plant cell that retains activity against VP8* antigen. In particular, the detection of anti-VP8* activity using an anti-SC detecting antibody is, to our knowledge, the strongest evidence provided so far in support for the formation of a fully human sIgA complex in the plant cell, as previous reports made use of hybrid murine IgG/A [10]. The presence of JC was not assayed, as it is technically challenging, due to the poor accessibility of the molecule and the lack of appropriate detecting antibodies. Therefore, the presence of incomplete complexes within the plant sIgA pool containing only SC bound to IgA in the absence of J-chain cannot be formally discarded. However, this is very unlikely, as it is well established that SC can only bind IgA when this is dimerized by the J-chain [35–37]. The small background signal observed when developing antigen-ELISA plates with anti-SC (Figure 2c) is probably due to un-specific binding of glycan structures within the SC, which are known to un-specifically bind to certain pathogens [38–40]. Also, VP8* has been previously described to bind glycans of the family of sialic acid [41,42]. However, plants lack this type of glycosylation, and therefore, it is unlikely that the observed reactivity is due to VP8* binding activity.

In addition, it was found that IgAα1-based designs presented better results than IgAα2 in all the combinations tested. This is somehow surprising, because the extended hinge of the IgAα1 isotype was expected to result in lower stability against protease degradation, therefore leading to lower activity levels [43]. However, it seems that a long hinge does not suppose a handicap for this specific antibody. The IgAα1 hinge is *O*-glycosylated in animal cells with mucin-like sugars, which is thought to confer additional proteolytic defense [44]. Mucin-like *O*-glycosylation cannot be achieved natively in plant cells. Instead, arabinose residues linked to hydroxyproline have been reported in the hinge of plant-made IgAα1 [45]. Whether plant-specific *O*-glycosylation patterns confer additional stability to IgAα1 remains to be elucidated [9].

Finally, a positive effect of ER retention in the overall anti-VP8* activity was clearly observed. It is well established that antibodies retained in plant ER by the addition of a KDEL signal accumulate at higher levels than antibodies that are secreted to the apoplast [21,22,25]. Interestingly, we observed that this effect is more noticeable when the KDEL signal is added to the SC. It is worthwhile to notice that the addition of KDEL to the SC increases both the anti-VP8* sIgA activity (as measured using anti-SC antibody), as well as the overall anti-VP8* IgA activity, as detected with anti-HC and anti-LC antibodies. This served as an additional indication that a significant portion of the anti-VP8* IgA pool is present in the form of a sIgA.

Further confirmation of the integrity of sIgA complex was obtained with the affinity purification of the resulting recombinant product. As shown, SC co-purifies with IgA when SSL7 is used in affinity binding experiments. SSL7 is reported to bind HCα between domains Cα2 and Cα3 [32,46], therefore discriminating between full-size IgA and Fab fragments. In the same conditions, recombinant SC alone was not recovered from SSL7 columns, indicating that the SC is unable to bind SSL7 on its own. Interestingly, the recovery rate of sIgA (as detected using an anti-SC antibody prior and after affinity purification) was lower than the recovery rate measured for total IgA (compare panels 1 and 2 with panel 3 in Figure 3c). A possible explanation is that only a fraction of the sIgAα1 complexes are covalently stabilized by disulfide bonds, whereas the remaining complexes are weakly kept together by non-covalent bonds, which can be broken apart during the purification process. Non-covalent complexes were previously described for native sIgAα2 [47], and therefore, it is plausible that non-covalent sIgAα1 complexes could occur in plants due to, e.g., partial assembly and/or inappropriate redox conditions in the plant ER. Although the molar ratio between SC and HC (1:4) could partially explain the low abundance of the SC band in purified IgA samples, the low recovery rate of sIgA compared to mIgA in SSL7 purification is probably contributing to these results.

Overall, it was established that maximum anti-VP8* activity was achieved by transient transformation of a multigene design comprising HCα1, LCλ, KDEL-tagged SC and JC. Using this combination, up to 32.5 μg of HC in its different IgA assembly forms was obtained per gram of fresh weight, with at least one third of this amount being present in the form of sIgA. The remaining two thirds of measurable IgA activity do not form secretory complexes. Free SC in relative large amounts has been detected in all sIgA combinations (data not shown), which would suggest that SC is not a limiting factor in sIgA formation. Thus, it is likely that the low level of complex formation is due to limiting JC expression. This could be resolved by placing the JC under the control of a stronger promoter and/or a stronger expression system.

Further improvements in terms of both the total IgA yield and the sIgA/mIgA ratio may be necessary for those applications involving high antibody doses. Moreover, additional considerations, such as the *N*- and *O*-glycosylation patterns of each antibody form in the context of passive mucosal immunotherapy, should be also considered [9]. Nevertheless, we think that the current production levels, combined with the high speed and combinatorial versatility of the described platform may provide sufficient competitive advantages for the production of monoclonal sIgAs in low-dose applications and/or in minimally processed formulations.

## 4. Experimental Section

### 4.1. Cloning and Assembly of Modular Pieces

The DNA sequences corresponding to the constant regions of human alpha1 heavy chain (HCα1), alpha2 heavy chain (HCα2), lambda light chain (LCλ) and secretory component (SC) were obtained from Open Biosystems, Huntsville, AL, USA. Kappa light chain (LCκ) and J-chain (JC) were codon optimized for *Solanum lycopersicum* and synthesized by GeneScript, NJ, USA (NCBI Accession numbers KC515402 and KC515401, respectively). The variable regions against VP8* were obtained from a scFv phage display, selected as described earlier [31]. The DNA module encoding the signal peptide for secretion was obtained by PCR from tomato SGN-U212775 unigene. Taking advantage of the property of seamless assembly, the junctions between the signal peptide and the coding sequences, e.g., HC (PSLLA-QVQLL), were tested with the signal P algorithm to ensure a correct processing into mature protein. PCR amplification was performed by using the Advantage-2 DNA Polymerase Mix (Clontech, Mountain View, CA, USA). The primers used for amplification of each basic part were synthesized by IDTdna, Coralville, IA, USA (Table S1). PCR was analyzed by agarose 1% gel electrophoresis and purified using the QIAquick PCR Purification Kit (Qiagen, Hilden, Germany). Amplified parts were TA cloned using the pGEM-T Easy Vector System (Promega, Madison, WI, USA), and 1 μL of the ligation was transformed into DH5α electrocompetent cells. Plasmid DNA preparations were obtained by using the E.Z.N.A. Plasmid Mini Kit I (Omega Bio-Tek, Norcross, GA, USA). Plasmid DNA concentration was measured using a Nano Drop Spectrophotometer 2000 (Thermo Scientific, Rockford, IL, USA). Positive clones were selected in ampicillin-containing plates and confirmed by plasmid restriction analysis (EcoRI, NotI from Thermo Fisher Scientific, Waltham, MA, USA) and by sequencing. Assembly reactions were performed basically as described by [28] using BsaI and BsmBI (New England Biolabs, Ipswich, MA, USA) as restriction enzymes in 25-cycle digestion/ligation reactions. T4 DNA ligase was purchased from Promega. One microliter of the reaction was transformed into DH5α electrocompetent cells. Positive clones were selected in kanamycin or spectinomycin-containing plates. Plasmid DNA preparations were made by using the E.Z.N.A. Plasmid Mini Kit I (Omega Bio-Tek). Plasmid DNA concentration was measured using a Nano Drop Spectrophotometer 2000 (Thermo Scientific). Constructs were confirmed by plasmid restriction analysis and by sequencing. Constructs for plant functional assays were transferred to *Agrobacterium tumefaciens* electrocompetent strain GV3101 containing pSoup plasmid.

### 4.2. Strains and Growth Conditions

*Escherichia coli* DH5α was used for gene cloning, and *Agrobacterium tumefaciens* strain GV3101 with pSoup was used for plant agroinfiltration and transformation experiments, as described in [28].

### 4.3. Plant Transient Transformation

Agroinfiltration was performed as previously described in [48]. Briefly, overnight grown bacterial cultures (5 mL) were sedimented by centrifugation (15 min, 3000× *g*), resuspended in agroinfiltration buffer (AB) (10 mM MES pH 5.6, 10 mM MgCl_2_, 200 μM acetosyringone) and incubated for 2 h at room temperature (RT) on a horizontal rolling mixer. Bacterial cultures were diluted in AB to an optical density of 0.2 at 600 nm. Co-infiltrations of each construct with both pGreen_P19 (bushy stunt virus-TBSV-P19, suppressor of silencing) [28] and pGreen_Luciferase (*Firefly Luciferase* Genetic reporter under the control of a Nopaline synthase promoter and terminator) were performed by mixing equal volumes of the corresponding bacterial suspensions. Inoculations were carried out by syringe-agroinfiltration in leaves of 4–5 weeks old *Nicotiana benthamiana* plants (growing conditions: 24 °C day/20 °C night in a 16 h light/8 h dark cycle). Samples were collected 5 days post-infiltration and examined for transgene expression.

### 4.4. Plant Material and Sample Preparation

For sample preparation, *Nicotiana benthamiana* leaves were ground with a mortar and a pestle to a fine powder under liquid nitrogen, and the total soluble protein (TSP) was extracted with 1:3 (*w:v*) phosphate buffer saline (PBS) pH = 7.4 complemented with 0.5 mM PMSF protease inhibitor. After mixing, the suspension was centrifuged twice at 2 °C at 16,000× *g*, and the supernatant, referred to as clarified crude extract, was immediately used for analysis. Samples were equalized with the luciferase reporter system as an internal standard. Luciferase activity was determined with the Dual-Luciferase Reporter Assay System (Promega), following the manufacturer’s procedures, and luminescence was measured with a Glomax 96 microplate luminometer (Promega).

### 4.5. VP8* Rotavirus Surface Protein Production

Recombinant VP8* was obtained as described in [31]. Briefly, *Escherichia coli* M11 were transformed with Plasmid pQEVP8*, kindly provided by Monedero from Instituto de Agroquímica y Tecnología de Alimentos (IATA, Valencia, Spain). Purification was performed using HisTrap HP affinity columns (GE Healthcare, Buckinghamshire, UK), following the manufacturer’s procedures.

### 4.6. ELISAs for the Quantification and Detection of VP8* Binding Activity of IgA and sIgA

Plates (Corning, New York, NY, USA) were coated overnight at 4 °C in coating buffer (50 mM carbonate buffer pH 9.8) either with anti-IgA capture antibody 1:500 (Sigma-Aldrich, St-Louis, MO, USA) for IgA/sIgA quantification or with 10 μg/mL of recombinant VP8* for the detection of VP8* binding activity. Plates were then washed four times in PBS and blocked with a 2% (*w*/*v*) solution of ECL AdvanceTM Blocking agent (GE Healthcare) in PBS-T [0.1% (*v*/*v*) Tween 20 in PBS]. Samples were diluted in PBS as required for each assay and incubated for 1 h at room temperature. After incubation, plates were washed four times in PBS, and the antibodies for detection were added in PBS-T-2% blocking buffer (GE Healthcare). Four different antibodies were used for detection in VP8*-ELISAs: anti-HC (HRP-conjugated) 1:5000 (Sigma Aldrich, St. Louis, MO, USA), anti-LCλ (non-conjugated) 1:5000 (Sigma Aldrich), anti-LCκ (non-conjugated) 1:5000 (Pierce Thermo Scientific) and anti-SC (non-conjugated) 1:500 (Gentaur, Kampenhout, Belgium). Anti-rabbit-HRP (GE Healthcare) secondary antibody (1:5000) was used after the non-conjugated detecting antibodies. For quantification, anti-HC (HRP-conjugated) 1:5000 (Sigma Aldrich) and anti-SC (non-conjugated) 1:500 (Gentaur, Kampenhout, Belgium) were used. Anti-rabbit-HRP (GE Healthcare) secondary antibody 1:5000 was also used for detection after the anti-SC detecting antibody. After four PBS washes, the substrate (*O*-phenylenediamine from Sigma Aldrich) was added, and the reactions were stopped with 3 M HCl. Absorbance was determined at 492 nm. The mean and SD of three samples of each combination were calculated for every VP8*-ELISA. A standard curve of HC content in a commercial IgA obtained from human colostrums (Sigma-Aldrich) was used to calculate the HC content in IgA/sIgA samples. The same standard was used to estimate the sIgA content in sIgA-containing samples using anti-SC as the secondary antibody. To facilitate comparisons, all antibody concentrations were provided as equivalents of HC. Three replicates were analyzed per each experimental point, and the mean ± SD was calculated.

### 4.7. SDS-PAGE and Western Blot Analysis

Proteins were separated by SDS/PAGE in 10% denaturing gels (Invitrogen, Carlsbad, NM, USA). Gel staining was carried out with coomassie following standard procedures. For Western blot analysis, blots were incubated with 1:20,000 anti-HC (Sigma Aldrich), 1:10,000 anti-LC (Sigma Aldrich) or anti-SC 1:5000 (Gentaur), followed by 1:20,000 μg/mL HRP-conjugated anti-rabbit IgG secondary antibody (GE Healthcare) for the detection of the LC and SC. Blots were developed with an ECL Plus Western Blotting Detection System (GE Healthcare).

### 4.8. SSL7 Affinity Purification

The SSL7 protein specifically binds the hydrophobic interface between the Cα2 and Cα3 domains of the Fc portion of the HC. Protein extracts, prepared as explained previously, were further clarified using a 0.22 μm Stericup (Millipore, Billerica, MA, USA) on ice. Purification steps were performed as previously described by [31].

## 5. Conclusions

We show here the feasibility of combinatorial optimization of sIgA production in plants using GoldenBraid DNA cloning technology. Moreover, we demonstrate that a fully human sIgA is assembled in the plant cell, and that the maximum yields of the anti-rotavirus sIgA2A1 used in this study are obtained when HCα1 and LCλ are used in combination with an ER-retained secretory component.

## Supplementary Information



## Figures and Tables

**Table 1:** Screening of 16 versions of sIgA by ELISA test. Anti VP8* binding activity is expressed in terms of Abs_492_ (Abs, absorbance).

	LCλ/SC	LCλ/SCkdel	LCκ/SC	LCκ/SCkdel
HCα1	++	++	−	−
HCα1kdel	+++	++	−	−
HCα2	++	++	−	−
HCα2kdel	++	++	−	−

+++ refers to Abs > 2.0

++ refers to Abs > 1.0

− refers to Abs < 0.3.

**Table 2:** IgA/sIgA levels, calculated as HC equivalents, from clarified crude extracts, referred to total soluble protein (TSP) and fresh weight (FW) of the two IgA and sIgA best performing combinations. Means plus or minus the standard deviation of three biological replicates are indicated for each section.

Construct	Best Performing Combination	Total IgA (%TSP)	Total IgA (μg/g FW)	sIgA (%TSP)	sIgA (μg/g FW)
IgA	HCα1kdel-LCλ	1.1 ± 0.1	31.6 ± 3.7	0	0
sIgA	HCα1-LCλ-JC-SCkdel	1.5 ± 0.1	32.5 ± 1.1	0.5 ± 0.1	11.0 ± 0.2
