# Supplementary material for: Combinatorial Analysis of Secretory Immunoglobulin A (sIgA) Expression in Plants
Source: Int J Mol Sci. 2013 Mar 18;14(3):6205–22. doi: 10.3390/ijms14036205 (PMC3634489; doi:10.3390/ijms14036205)
Supplement: Supplementary file 1 [file ijms-14-06205-s001.docx]

Supplementary Information

**Table S1.** Oligo List.

| **BASIC PART** | **PRIMER NAME** | **PRIMER SEQUENCE 5′-3′** |  |
| --- | --- | --- | --- |
| 35s | | A10JUL09 | CCCGTCTCACCACACTAGAGCCAAGCTGATCTC |
|  |  | A10MAY111 | CCCGTCTCACATCGATATCACTAGTGCGGCCGC |
| SP | | A10SEP05 | CCCGTCTCAGATGGGCACTTCCTCTGTTTT |
|  |  | A10JUL10 | CCCGTCTCAGGCAAGGAGGGACGGGAGAAGGAG |
| VH | | A10JUL16 | CCCGTCTCATGCCCAGGTGCAGCTGTTGCAGT |
|  |  | A10JUL17 | CCCGTCTCAATGCGACGGTGACCAGGCTACCTT |
| VL | | A10JUL11 | CCGTCTCATGCCTCTTCTGAGCTGACTCAGGA |
|  |  | A10JUL12 | CCGTCTCAACCTAGGACGGTCAGCTTGGTCC |
| CLλ | | A1JUL13 | CCCGTCTCAAGGTGGTCAACCAAAGGCCGCCCCCTCTGTCACTCT |
|  |  | A10JUL03 | GGCGGGAGTGGAGACAACCAAACCC |
|  |  | A10JUL04 | GGGTTTGGTTGTCTCCACTCCCGCC |
|  |  | A10JUL14 | CCCGTCTCACTCATGAACATTCTGTAGGGGCCA |
|  |  | A10JUL14KDEL | CCCGTCTCACTCAAAGTTCATCTTTTGAACATTCTGTAGGGGCCA |
| CH1 | | A10JUL18 | CCCGTCTCAGCATCCCCGACCAGCCCCAAGGTC |
|  |  | A10JUL19 | CCCGTCTCACTCAGTAGCAGGTGCCGTCCACCT |
|  |  | A10JUL20 | CCCGTCTCACTCAGAGTTCGTCCTTTGAACATTCTGTAGGGGCCA |
| CH2 | | A10JUL18 | CCCGTCTCAGCATCCCCGACCAGCCCCAAGGTC |
|  |  | A10AGO01 | ACCATGGGGAAACCTTCACC |
|  |  | A10AGO02 | TGCAGGTGAAGGTTTCCCCATGGTTCCATGG |
|  |  | A10JUL19 | CCCGTCTCACTCAGTAGCAGGTGCCGTCCACCT |
|  |  | A10JUL20 | CCCGTCTCACTCAGAGTTCGTCCTTTGAACATTCTGTAGGGGCCA |
| SC | | A10SEP07 | GGGGTCTCATCATCGTCTCATGCCAAGAGTCCCATATTTGGTCC |
|  |  | A10SEP08 | GCATATTTGCTGGAAACGTAGC |
|  |  | A10SEP09 | TCCTCGGAGGGCTACGTTTC |
|  |  | A10SEP11 | GGGGTCTACTCACCTGGGATCCTGAATGGCTTTGT |
|  |  | A10SEP10 | GGGGTCTCACTCAGAGTTCGTCCTTCCTGGGATCCTGAATGGCTT |
| Tnos | | A10ABR03 | CCCGTCTCATGAGGGAATGGATCTTCGATCCCGACGT |
|  |  | A10JUL15 | CCCGTCTCAGACGGCGAGTCGGTCCCATTATTGA |

© 2013 by the authors; licensee MDPI, Basel, Switzerland. This article is an open access article distributed under the terms and conditions of the Creative Commons Attribution license (http://creativecommons.org/licenses/by/3.0/).
